# Lung cancer risk and its potential association with PM_2.5_ in Bagmati province, Nepal—A spatiotemporal study from 2012 to 2021

**DOI:** 10.3389/fpubh.2024.1490973

**Published:** 2024-12-16

**Authors:** Basanta Kumar Neupane, Bipin Kumar Acharya, Chunxiang Cao, Min Xu, Pornpimol Kodsup Taylor, Shaohua Wang, Yujie Yang

**Affiliations:** ^1^State Key Laboratory of Remote Sensing Science, Aerospace Information Research Institute, Chinese Academy of Sciences, Beijing, China; ^2^University of Chinese Academy of Sciences, Beijing, China; ^3^Nepal Open University, Lalitpur, Nepal; ^4^Nepal Geographical Society, Kathmandu, Nepal; ^5^Taylor Foundation for the Arts and Sciences, Atlanta, GA, United States; ^6^Tulane University, New Orleans, LA, United States

**Keywords:** lung cancer, spatiotemporal distribution, PM_2.5_, pollution, Bagmati province

## Abstract

**Background:**

Despite examining the role of an association between particulate matter and lung cancer in low-income countries, studies on the association between long-term exposure to particulate matter and lung cancer risk are still contradictory. This study investigates the spatiotemporal distribution patterns of lung cancer incidence and potential association with particulate matter (PM_2.5_) in Bagmati province, Nepal.

**Methods:**

We performed a spatiotemporal study to analyze the LC – PM_2.5_ association, using LC and annual mean PM_2.5_ concentration data from 2012 to 2021. The study assessed the global spatial autocorrelation test using global Moran's I, applied hotspot analysis. A bivariate statistical analysis was performed to evaluate the association, we also applied the geographically weighted regression model (GWR) to look for possible relationships.

**Results:**

The annual mean crude incidence rate (CIR) and standardized incidence rate (SIR) were 5.16, and 6.09 respectively. The study reveals an increasing trend with notable municipal-level spatial variations. Bhaktapur municipality exhibits the highest CIR (243.88), followed by Panchkhal and Sunapati. Males consistently exhibit higher rates, particularly in middle-aged and older adult populations. Bhaktapur displayed the highest CIR in males (171.9) but very low in females (72). The spatial analysis identified concentration trends and hotspots developed in the Bhaktapur, Panchkhal, and Sunapati municipalities. The SIR showed fluctuating patterns of continuous rise until 2019, decrease in 2020, and rise again thereafter. Similar fluctuation association patterns were observed with PM_2.5_, the *r*-squared value consistently fluctuated during the study period.

**Conclusion:**

In this study, we found an association between PM_2.5_ exposure and lung cancer incidence. The findings underscore the need for targeted public health interventions, highlighting the role of PM_2.5_. Future research is suggested to explore the relationship between lung cancer distribution and various environmental risk factors for effective control and prevention. Addressing air pollution could potentially reduce future lung cancer risk.

## Introduction

Cancer is a major global health problem and rapidly increasing in the global south (1, 2). According to the World Cancer Report, lung cancer is one of the major causes of death in the world (3). In 2020, ~2.21 million new cases were identified, and 1.8 million people died due to lung cancer. For men, lung cancer is the most common cancer worldwide (4), with the adjusted standardized incidence rate (ASIR) and adjusted standardized mortality rate (ASMR) at 22.4 and 18.0 per 100,000, respectively. The study in Shandong province found that four health endpoints related to PM_2.5_ in males were higher than in females, and the health consequences of PM2.5 pollution appeared to be more severe in males compared to females (5). With increasing easy access to tobacco, pollution, and industrialization, lung cancer risk is rising in the global south continuously (6). Lung cancer risk distributed heterogeneously, depends on various geographical locations, mainly because of several risk factors, such as pollution concentration, people's lifestyle, smoking prevalence, topographic variation, and dietary habits (7).

According to the International Agency for Research on Cancer (IARC), 2,431 new lung cancer cases with 9.3 adjusted standardized incidence rates were reported in Nepal. A study in Nepal reported the ASIR and ASMR were 80.9 and 54.8 per 100,000, respectively, which is very high; it is visible that Nepalese people are vulnerable to cancer (8).

Cancer mortality rates are notably higher among men in Nepal's urban areas, with peak lung cancer incidence observed in the 70–74 age group (62). Lung cancer leads as the most common cancer in Nepal, with the crude incidence rate (CIR) rising significantly between 2003 and 2012, from 2.25 to 4.45 per 100,000 in males and from 1.52 to 2.85 in females (9). This type of cancer accounts for the highest number of cases in men and the third highest in women, representing the primary cancer-related fatality risk for males. In 2012, the incidence rate was recorded at 5.74 in males and 3.89 in females (9). The Nepal Health Research Council (NHRC) reported a lung cancer mortality rate of 41.6 per 100,000, with evidence indicating a gradual increase in recent years (10). The ASIR in Kathmandu is 18.1 for males and 10.4 for females, contrasting with lower rates in rural areas such as Rukum (13.2 in males and 5.2 in females) (62). Additionally, studies indicate a higher burden of lung cancer among males compared to females in Nepal (11, 52).

Lung cancer risk is known to be linked to pollution levels and population density. Studies such as Liao et al. (53) have demonstrated an association between PM_2.5_ concentration and lung cancer mortality, with multiple spatiotemporal studies identifying fine particulate matter (PM_2.5_) as a significant effect on human activities (12) and a contributor to lung cancer incidence (13, 14, 54). PM_2.5_ ranks among the top public health risk factors globally, linked to 4.58 million deaths annually (55). Nepal's air quality was ranked 162 out of 200 countries by the Environmental Performance Index (56), with Kathmandu among South Asia's most polluted cities. PM2.5 is considered a major pollutant, which has been associated with harming public health, and premature deaths contributed by LC (15). WHO reports indicate an average PM_2.5_ level of 140 μg/m3 in Nepal—ten times above the recommended limit (3). In Bagmati Province, high population density in urban areas increases outdoor activity exposure, elevating lung cancer risk among residents.

Studies suggest a unique relationship between environmental factors, geography, and disease patterns, highlighting the importance of spatially focused approaches for disease control and prevention (50). Geospatial technology is instrumental in epidemiological research, offering crucial insights into disease distribution and contributing to targeted intervention strategies (51). This study, therefore, aims to fill a significant research gap by examining the relationship between PM_2.5_ exposure and lung cancer incidence in Nepal's Bagmati Province, an area where spatial epidemiology studies are scarce, especially regarding lung cancer risk in the Himalayan context. We hypothesize that higher PM_2.5_ exposure correlates with elevated lung cancer incidence, and we explore this association across the province's lowest administrative levels. By identifying correlations and hotspot areas with significantly higher lung cancer trends, this research provides a foundation for more precise public health interventions and further studies into the environmental determinants of lung cancer in Bagmati Province.

## Methods

### Study area

**Figure 1:**
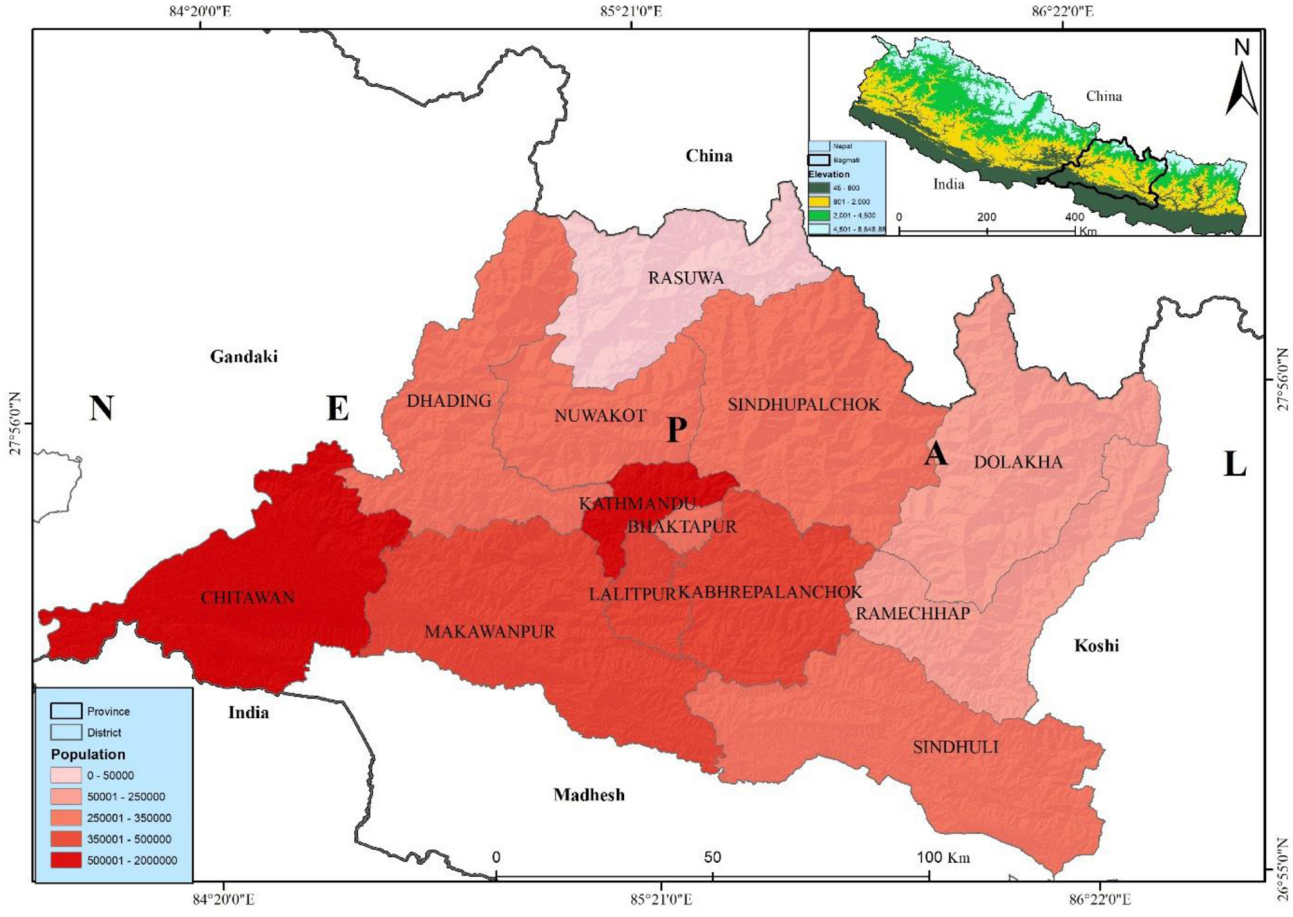
Location map and population distribution of study area.

Bagmati province, is most populated province, is situated in the central part of Nepal with latitudes 26°55′-28°23′N and longitudes 83°55′-86°34′E. It has an area of 20,300 sq. km, which is 13.79% of the country's total area. Among the total land, 27.29% is covered by forest. Bagmati province is bordered by Madhesh province and India in the south, China in the north, Gandaki province in the west, and Koshi province in the east (Figure 1). According to the administrative division, it has 13 districts, and 119 local levelcomposed of three metropolitan cities, one sub-metropolitan city, 41 municipalities, and 74 rural municipalities with 1,121 wards. Bagmati province has a 6,116,866 population, 3,068,182 females, and 3,048,684 males (16). The Bagmati province has the second-highest population in Nepal. Kathmandu is the capital city of Nepal, which is highly populated, with a population of about 2,041,578 in the district itself—believed to be ~3.1 million in the entire Kathmandu valley.

Bagmati Province, encompassing Nepal's capital (Kathmandu Valley), is the country's most populous and developed region, with high urban density and considerable migration flows that contribute significantly to elevated air pollution levels (57, 58). This combination of dense urban congestion and limited resources to support healthy living conditions has been linked to an increased risk of lung cancer, particularly in major cities (59). Non-communicable diseases (NCDs) are also on the rise, posing a growing public health concern (60). As Nepal continues to develop, the rising incidence of lung cancer has emerged as a critical challenge, significantly impacting public health in urbanized areas.

### Lung cancer data

Lung cancer data between 2012 and 2021 were collected from the Bhaktapur Cancer Hospital (BCH), one of the major government cancer hospitals in Bhaktapur city. The lung cancer data contains attributes including age, sex, address, and diagnosis date. The patient's permanent address is linked with the lung cancer data with a municipal map of Nepal in an ArcGIS environment. Out of 2,279 lung cancer cases, 95 were dropped due to missing information about the municipality, while the remaining 2,184 were successfully joined for further analysis. The municipality's age-specific populations were extracted from the NSO reports published by the Nepal government. The study used the municipality boundary map from the Department of Survey, Government of Nepal.

### PM_2.5_ data

The study utilized annual mean PM_2.5_ concentration data from the Socioeconomic Data and Applications Center (SEDAC) dataset spanning 2012 to 2019. This study used particulate matter concentration data from 2012 to 2019. This dataset has included PM_2.5_ and its spatial distribution map with 0.1° × 0.1° resolutions (https://sedac.ciesin.columbia.edu/citations-db) (17). The dataset extracted PM_2.5_ data from the Global Annual PM_2.5_ Grids, MODIS, MISR and SeaWiFS Aerosol Optical Depth (AOD) with GWR dataset released by NASA (https://sedac.ciesin.columbia.edu/data/set/sdei-global-annual-gwr-pm_2 − 5_-modis-misr-seawifs-aod-v4-gl-03/data-download) annual-gwr-pm_2.5_-modis-misr-seawifs-aod) with a resolution of ~1 × 1 km. This dataset is almost similar to ground-monitored PM_2.5_ data with *R*^2^ = 0.81 and has been used in several studies (18, 19). Then, we calculated the annual 2012–2021 PM_2.5_ mean concentration for each municipality at the centroids of these polygons using a linear regression model. The same method has been applied in a previous study by van Donkelaar et al. (20). Finally, the study linked lung cancer data and PM_2.5_ from 2012 to 2021 for each of the 119 municipalities of Bagmati province.

### Statistical analysis

For spatial statistical analysis, crude incidence rate (CIR) and age-adjusted standardized incidence rate (SIR) were calculated using the annual incidence rate. We used the national population of 2011 to calculate. Both rates were expressed per 100,000. In addition, to the Bagmati province, we also calculated CIR and SIR for Kathmandu Valley. The annual crude incidence rate was calculated by using the following equation:


(1) $$\begin{array}{l} \text{Crude incidence rate }\left(\text{CIR}\right)=\frac{\left(\Sigma C\right)}{\left(\Sigma P\right)}\times 100000 \end{array}$$

Where, C = Total lung cancer cases

P = Total population

The age-adjusted incidence rate (SIR) was calculated e with a standard age structure. We used 18 specific age groups (i.e., 0–4 years, 5–9 years, . . . 85 years or older) to calculate it. The study used the Poisson model to examine the statistical significance, and statistical significance was defined as two-tailed *p*-values < 0.05 for this study (21), it has been used to compare and understand the temporal distribution (22). The standardization rate is in practice and suitable for comparing incidence rates in different years. First, we calculated the age-specific incidence rate then we standardized it with the national standard population. The annual age-standardized incidence rate was calculated by using the following equation:


(2) $$\begin{array}{c} \text{Age}-\text{specific standardized incidence rate}\\ =\frac{\sum \frac{c_{i}}{y_{i}}\times s_{i}}{\sum S}\times 100000\;for\;i=1,2,3,\ldots \ldots n \end{array}$$

Where, ci = Number of new lung cancer cases in the ith age group

Si = Standard population of the ith age group

yi = Risk population of the ith age group

S = Total standard population

*n* = the number of age groups (*n* = 18)

### Spatial analysis

#### Crude incidence rate

Spatial analyses were conducted using GeoDa 1.6.7 and ArcGIS 10.5 (Esri Inc., California, USA) software (23). In GeoDa, we created a spatial weight file with a neighbored structure based on the K-nearest neighborhood criteria (five municipalities in this research), which was then imported to create spatially smoothed distribution maps. We computed the municipal-level CIR. The study employed a GIS mapping environment to visualize the lung cancer incidence rate over a decade. The study calculated the mean annual incidence rate using the GeoDa software by dividing the total number of lung cancer cases by the corresponding municipal population. The crude incidence municipal-level annual rates, CIR_LC_ for the ith municipal in the year t was estimated as:


(3) $$\begin{array}{l} {\text{CIR}}_{it}=\frac{1}{10}\left(\frac{X_{LC_{it}}}{P_{it}}\times 100000\right) \end{array}$$

where X_LCit_ represents the reported LC case counts at the municipality i (*i* = 1, 2, …, *n* = 2,184) for the year *t* (*t* = 2012, 2013, …, 2021), and P_it_ represents the population in municipality i for the year t.

#### Excess risk and rate

We also calculated excess risk to determine the lung cancer risk in Bagmati province. The excess risk can be determined by dividing the observed incidence in each municipality by the average incidence in all endemic locations. We calculated the excess rate for each municipality dividing total cancer cases by the corresponding municipal population each year. In the excess risk, value one is commonly used as a cut-off value, with less than one indicating lower incidence than predicted and more than one indicating higher incidence than expected. The municipal-level annual excess rate (ER)_LC_ for the ith municipal in the year t was estimated as:


(4) $$\begin{array}{l} ER_{it}=\frac{1}{10}\left[\overset{y_{2021}}{\underset{y_{2012}}{\sum }}\left(\frac{L{C\left(X\right)}_{it}}{\left(Y\right)Pop_{it}^{n}}\right)\times 100000\right] \end{array}$$

Where: LC(X) is the municipal level cases reported from the hospital (i) for the year (t), and (Y) is the population of the municipality (i) for the year (t) from 2012 to 2021, and census 2021 population is used for this calculation.

The study calculated a 10-year annual raw and excess risk map, which was subject to spatial autocorrelation analysis. Consequently, this map may not be accurately present the real distribution of lung cancer in the study area (24). To address this, we processed 10-year averaged incidence rates to generate a spatially smoothed lung cancer distribution map by correcting for spatial autocorrelation. We utilized the spatial empirical bayes method available in GeoDa for this purpose. Initially, we created a spatial weight file in GeoDa incorporating neighboring structures based on the K-nearest neighborhood criterion (five municipalities in this instance). This file was subsequently employed to produce the spatially smoothed distribution maps.

**Figure 2:**
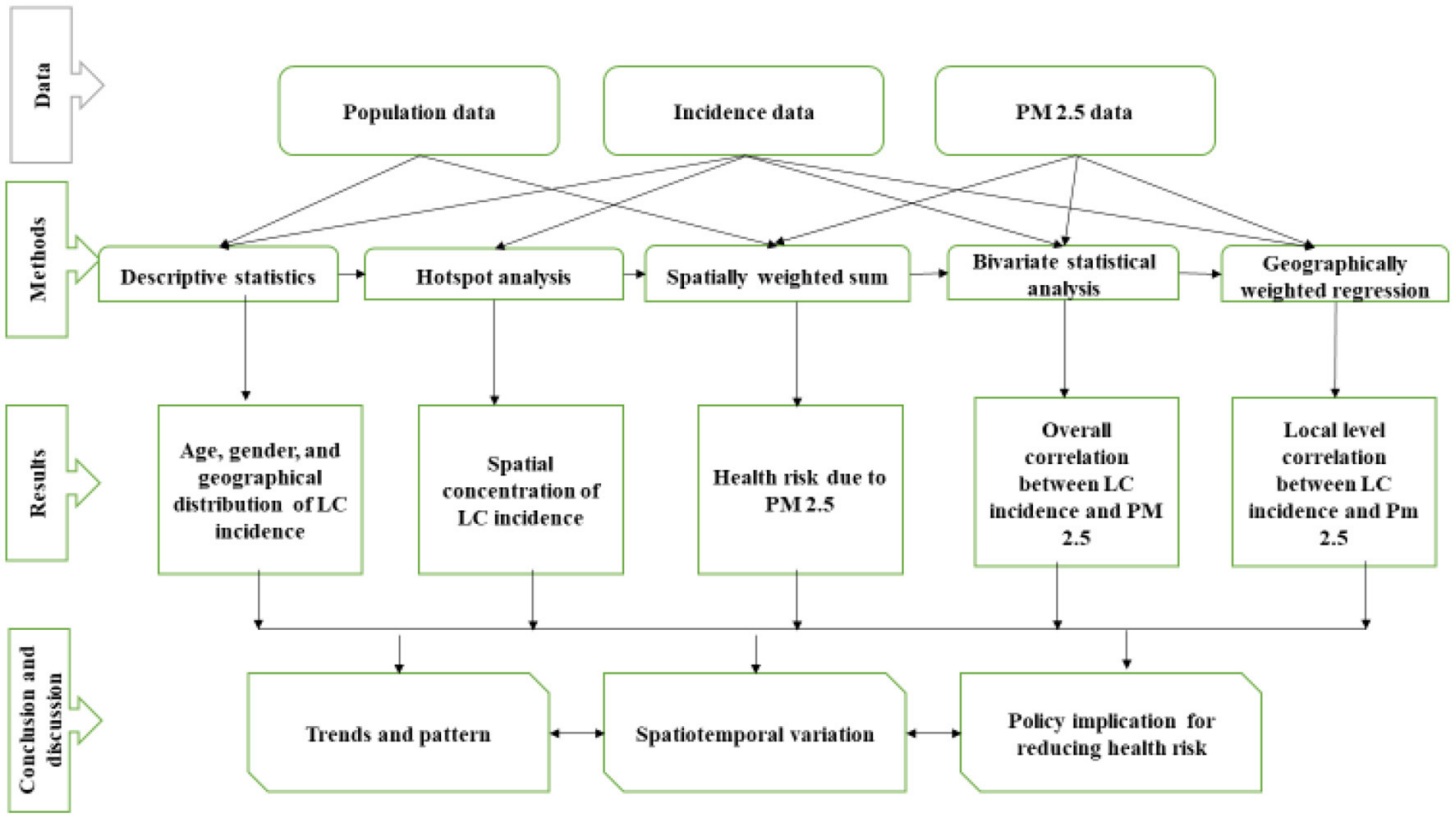
The overall research framework of the study.

The study assessed the global spatial autocorrelation test using global Moran's I (25) and then applied hotspot analysis, spatially-weighted sum, bivariate statistical analysis, and geographically-weighted regression (GWR) model (22). As shown in the methodological framework (Figure 2), we first calculated the hotspot analysis based on municipal-level incidence rate to detect the geographical patterns of lung cancer incidence. After the hotspot analysis, we calculated the spatial weighted sum, applied to population-level risks of different municipalities by overlapping the population density (22). The following two stages attempted to explore the spatiotemporal correlations between PM_2.5_ distributions and lung cancer risk. A bivariate statistical analysis was performed to evaluate the association, while the geographically weighted regression model (GWR) was applied to look deeply into the possible relationships between different municipalities. A GWR model was used to examine the spatially varying association between lung cancer and PM_2.5_; it is shown with the standard deviations of the coefficient of determination (*R*^2^) at different municipalities. The study examined the GWR after applying the *t*-test to the linear regression.

The study used the Getis–Ord G^*^ statistic, a scoring system derived from a significance test, to calculate whether features with high or low values tended to cluster within the study area. This software detects statistically relevant hotspots surrounded by other features with high values for each component in a dataset (26). The *Z*-score demonstrates the spatial clustering of features with high or low values. The formula is given below:


(5) $$\begin{array}{l} G_{i}^{*}=\frac{\sum_{j=1}^{n}w_{i,j}\;x_{j\;-\;}\bar{X}\sum_{j=1}^{n}w_{i,j}}{S\sqrt{\left[\frac{n\sum_{j=1}^{n}w_{i,j}^{2}-{\left(\sum_{J=1}^{n}w_{i,j}\right)}^{2}}{n-1}\right]}} \end{array}$$

Where x_j_ is the value for j, w_ij_ is the value of spatial value between I and j. n is equal to the total number of features and:


(6) $$\begin{array}{l} \bar{X}=\frac{\sum_{j=1}^{n}x_{j}}{n} \end{array}$$

(7) $$\begin{array}{l} s=\sqrt{\frac{\sum_{j=1}^{n}x_{j}^{2}}{n}-{\left(\bar{X}\right)}^{2}} \end{array}$$

The $G_{i}^{*}$ value is the *Z*-score, so no need for additional calculation.

Then, we used the spatially-weighted sum tools to examine the health risks from particulate matter (14, 22), previous research adopted the classical approach to present the interactive outcomes of more factors by adding the value of the elements from one layer to another layer with area attributes (27). The study had calculated the lung cancer risk based on population density and we overlay annual PM_2.5_ with the population density map on the ArcGIS environment. The study used the population density and PM_2.5_ raster data to examine and identify the risk using overlap analysis.

The spatially weighted sum was calculated by overlapping the population densities and distributions of PM_2.5_ to calculate the health risks. This study used the Jenks natural breaks default in ArcGIS in this study. Natural break classes are based on natural groupings inherent in the data. Class breaks are identified as the best group with similar values and maximize class differences. The output is categorized into five levels and spatially weighted to show the population-level health risks at different municipalities.

Following hotspot analysis, we computed bivariate statistical analysis to examine the spatial interrelation between PM_2.5_ and lung cancer incidence (28). This method is accessible for exploring relationships between different types of attribute data. We transferred the polygon data into the raster data before starting the spatial analysis. The polygon data were transferred at a 30 m resolution to raster data for spatial calculation and analysis in ArcGIS.

Finally, we employed the GWR model to analyze the spatially heterogeneous association of lung cancer with PM_2.5_. The GWR model fits the regression of each location and reports mapable regression parameters including the coefficient of determination i.e., *R*-squared assuming the relationship may vary in different locations (13). The following is the equation for a typical GWR of the ordinary least-square regression (OLS) model:


(8) $$\begin{array}{l} Y_{i}\left(u\right)=\beta_{o_{i}}\left(u\right)+\beta_{1i}\left(u\right)x_{1i}+\beta_{2i}\left(u\right)x_{2i}+\ldots +\beta_{m_{i}}\left(u\right)x_{mi} \end{array}$$

Where y is the lung cancer incidence rate of each municipality while x represents the environmental exposure i.e., PM_2.5_ in our case. The β_0i_ indicates the location of the study unit.

Based on the geographical distribution of lung cancer incidences and health risks associated with PM_2.5_, our research investigated the spatial correlations between these health risks. Initially, a bivariate statistical analysis examined the overall spatial correlation between raster layers representing the distribution of PM_2.5_ and lung cancer incidence.

## Results

Two thousand one hundred and eighty-four lung cancer cases were reported to the Bhaktapur Cancer Hospital during the study period. Among these, 1,315 cases were males, 867 females, and, 1 third gender. The annual mean CIR was 5.16. During the study period, the minimum CIR (3.02) was reported in 2012 while the maximum CIR (6.85) was reported in 2019 (Table 1). In the same period, the mean CIR of Kathmandu Valley was 5.53, the minimum was reported in 2012 and the maximum was reported in 2021. The minimum and maximum CIR were 3.86 and 6.84, respectively. Likewise, the age-standardized rates were calculated using the Nepal Census 2011. The annual mean SIR in Bagmati Province was 6.09. The minimum SIR, reported in 2012, was 3.58, while the maximum SIR, reported in 2019, was 8.09. The annual mean SIR in Kathmandu Valley was 6.56. The lowest SIR, 4.96, was observed in 2012, while the highest SIR, 7.81, was observed in 2021.

**Table 1:** Lung cancer incidence in Bagmati province and Kathmandu Valley (KV).

**Year**	**Kathmandu Valley (KV)**			**Bagmati province**		
	**No. of cases**	**Crude rate**	**SIR**	**No. of cases**	**Crude rate**	**SIR**
2012	74	3.86	4.96	119	3.02	3.58
2013	82	4.28	5.43	148	3.7	4.38
2014	100	5.22	5.56	182	4.49	5.3
2015	116	6.06	7.47	207	5.05	5.96
2016	122	6.37	7.72	246	5.91	6.97
2017	101	5.27	6.28	237	5.62	6.62
2018	113	5.90	7.05	252	5.89	6.93
2019	122	6.37	7.42	297	6.85	8.09
2020	98	5.12	5.91	221	5.03	5.91
2021	131	6.84	7.81	271	6.09	7.17

**Figure 3:**
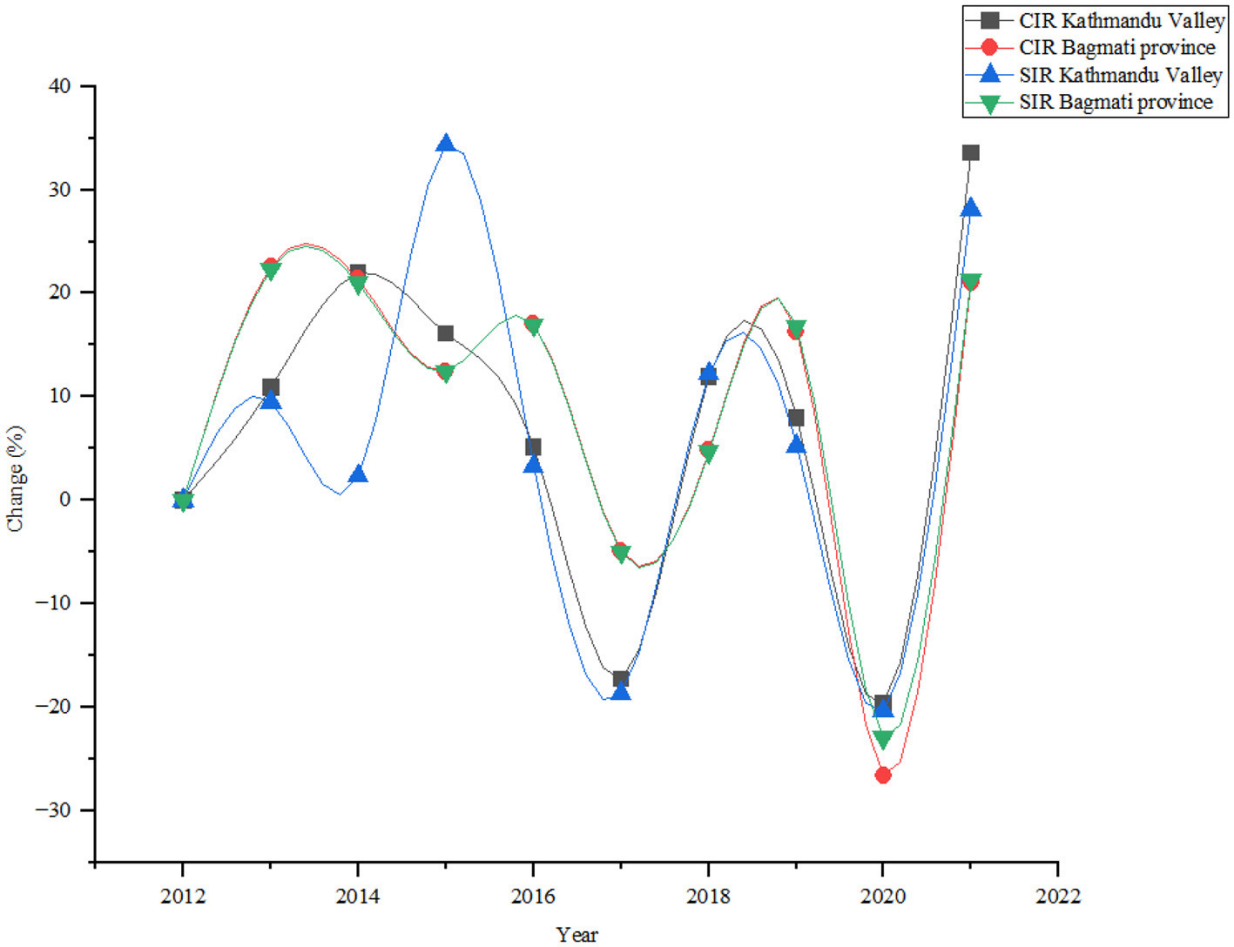
Annual change during the study period.

The annual analysis demonstrated several notable trends and fluctuations during the study periods (Figure 3). The number of lung cancer cases increased steadily from 74 in 2012 to 131 in 2021, with a notable increase of 77.03% in Kathmandu Valley. The crude rate, rises from 3.86 in 2012 to 6.84 in 2021, with an increment of 77.20%. In Bagmati province lung cancer cases increased from 119 in 2012 to 271 in 2021, showing a significant increase of 127.73%. The study found the crude rate rose from 3.02 in 2012 to 6.09 in 2021, showing an increase of 101.66% during the study period.

**Figure 4:**
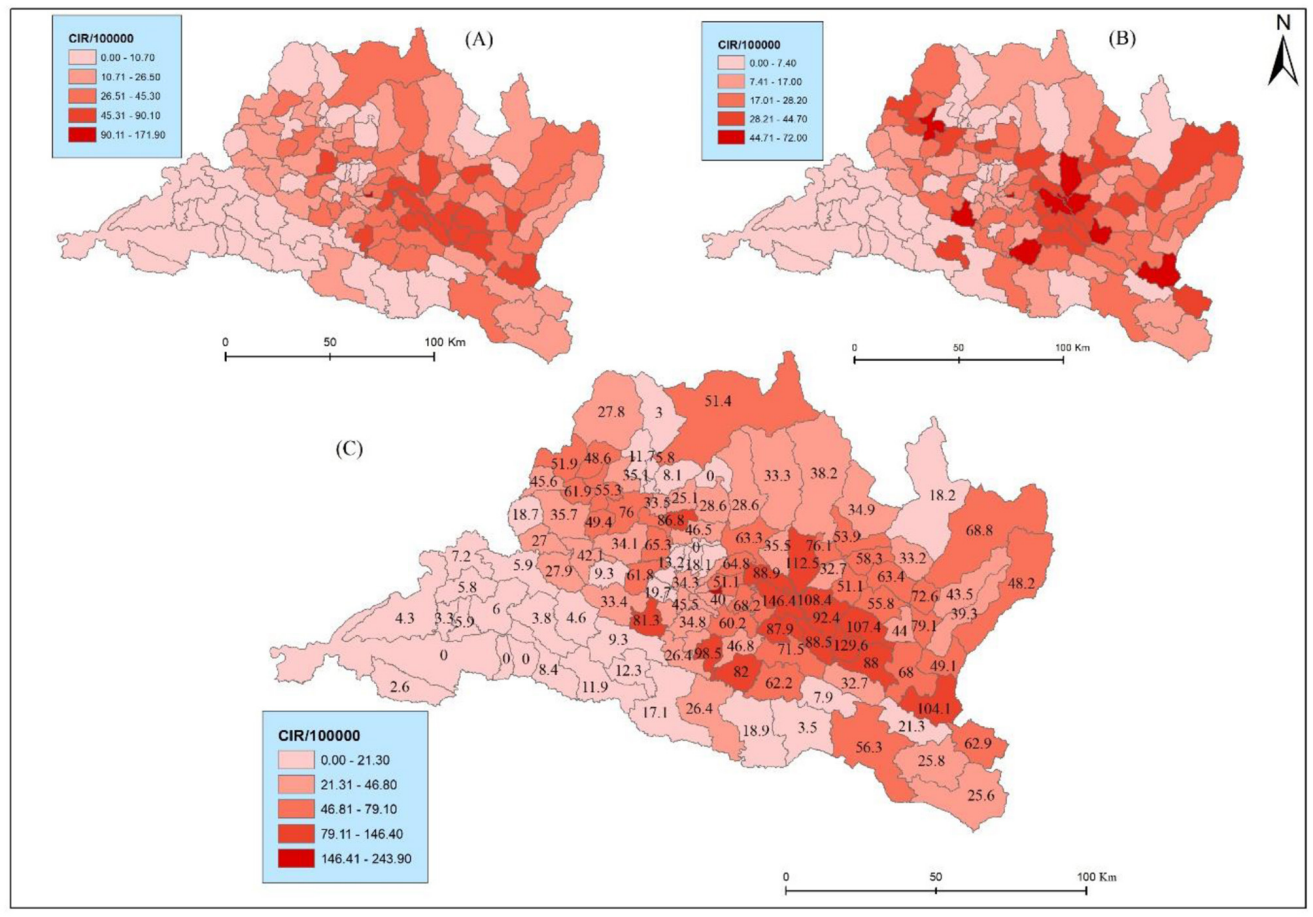
**(A)** CIR Male, **(B)** CIR Female, **(C)** CIR in Bagmati province.

The results reveal marked spatial variation and heterogeneity in the distribution of CIR. Bhaktapur municipality exhibits the highest CIR (243.88), followed by Panchkhal and Sunapati with 146.39 and 129.62, respectively (Figure 4C). Bhaktapur municipality was the only municipality in the Kathmandu Valley with higher health risks. Conversely, municipalities such as Raksirang and Marin reported relatively low CIR at 3.85 and 3.47, respectively. Lung cancer cases are reported in higher numbers from Kathmandu, Lalitpur, and Bhaktapur, but due to the high density of the population, they had a low crude incidence rate. This detailed breakdown underscores the heterogeneity in lung cancer burden across municipalities, guiding targeted public health interventions and resource allocation based on specific incidence patterns. Specifically, within the province, the municipalities of Bhaktapur (one), Lalitpur (one), Ramechhap (three), Kabhrepalanchwok (six), Sindhupalchok (two), Nuwakot (one), and Makawanpur (one) exhibit a notable presence lung cancer. This distribution of CIR underscores the need for further investigation and targeted public health interventions in these identified areas to mitigate potential health challenges and improve overall community wellbeing.

The results further show spatial variations of CIR in terms of sex (Figures 4A, B). For instance, Bhaktapur displayed the highest CIR in males (171.9) but very low in females (72), The same pattern was found in another Panchkhal municipality of Kabhrepalanchwok (90.1) and females (56.3). Notably, Indrasarowar rural municipality exhibited a significantly higher CIR on females (51.7) compared to males (29.6). Further, the Meghang rural municipality of Nuwakot showed a higher CIR for females (47.4) compared to males (7.9).

**Figure 5:**
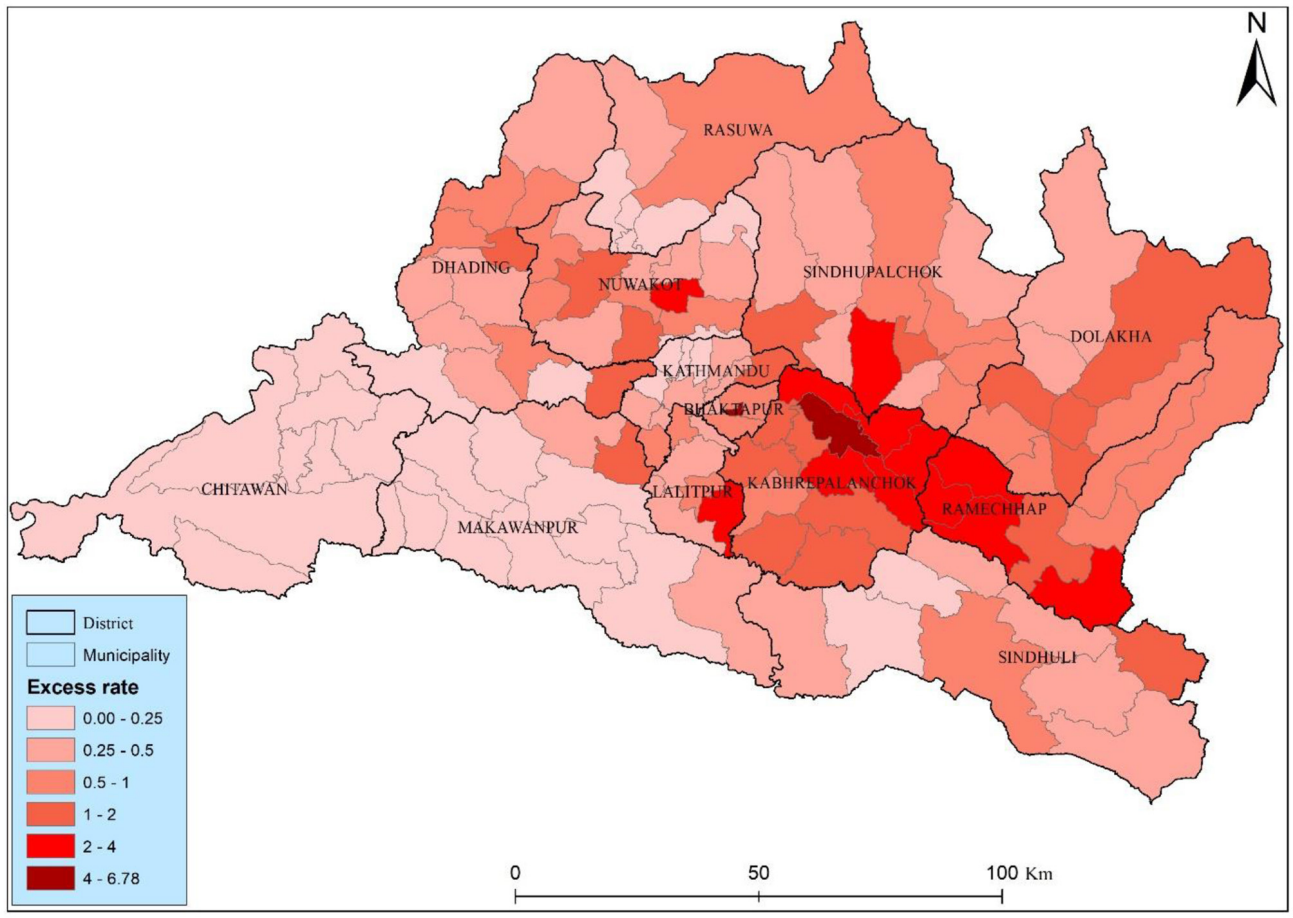
Excess risk map of the Bagmati province.

The study found twenty-one municipalities with an excess risk higher than two, suggesting elevated risk relative to the municipality average (Figure 5). Two municipality Bhaktapur and Panchkhal had the highest excess risk among the other municipality, they had 6.78 and 4.78, respectively. The high level risk was distributed close to the Kabhrepalanchok, Sindhupalchwok, and Ramechhap districts. Most of the municipalities (73) has a moderate health risks, those municipalities have 0.5–2 excess risk, particularly concerning lung cancer, are observed across various municipalities in the Bagmati province of Nepal. The distribution of health risk of the study area underscores the detail investigation and started to lung cancer interventions to mitigate potential lung cancer risk.

**Figure 6:**
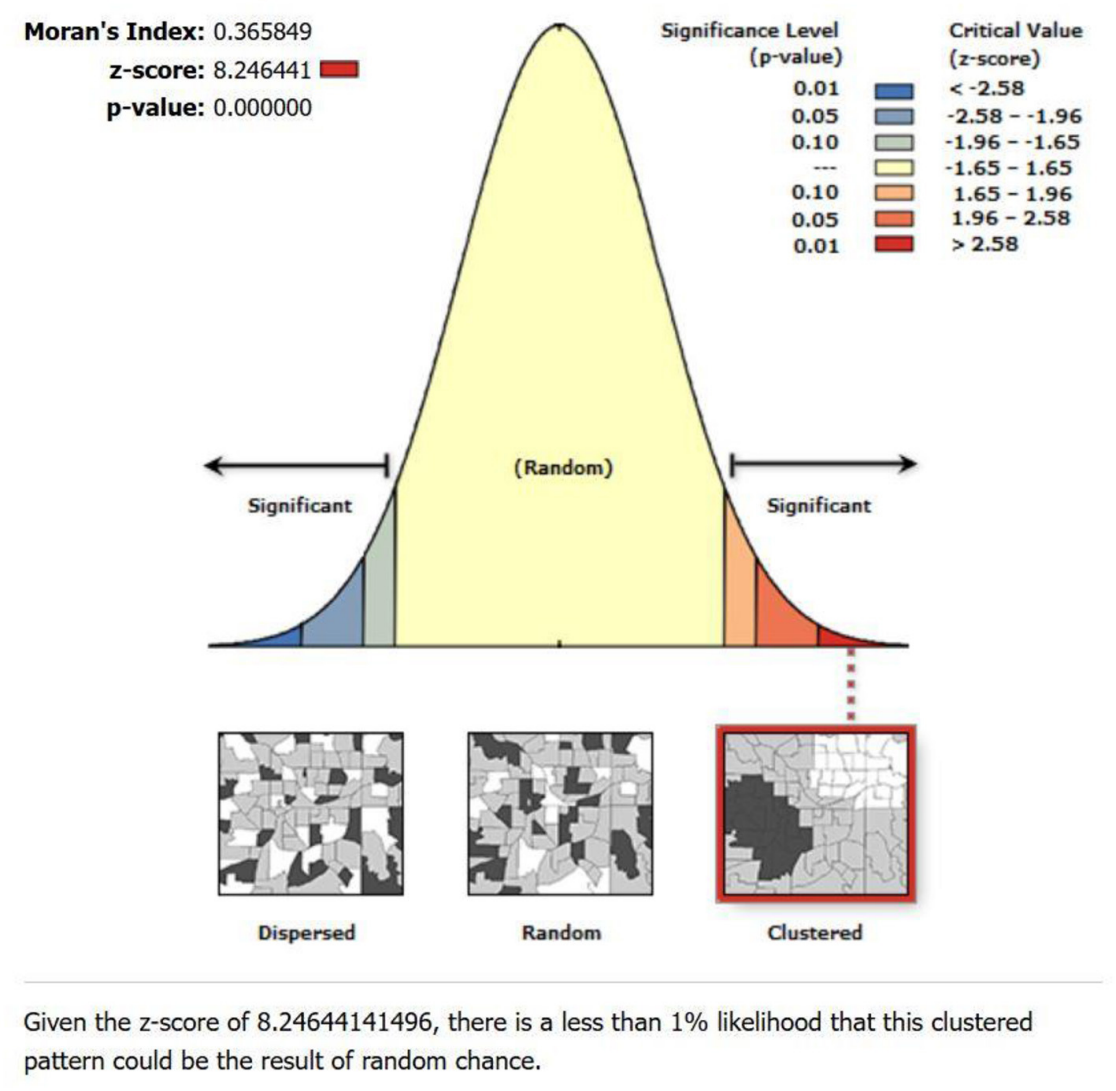
Spatial autocorrelation test report.

The global spatial autocorrelation test using Moran's I showed positive spatial autocorrelation with Moran's *I*-value of 0.366, a *Z*-score of 8.25, and a *p*-value of 0.000 (Figure 6). This finding suggests a non-random distribution of lung cancer incidence rates within the study area. The positive Moran's *I* value indicates positive spatial autocorrelation, implying that neighboring municipalities tend to have similar lung cancer incidence rates.

**Figure 7:**
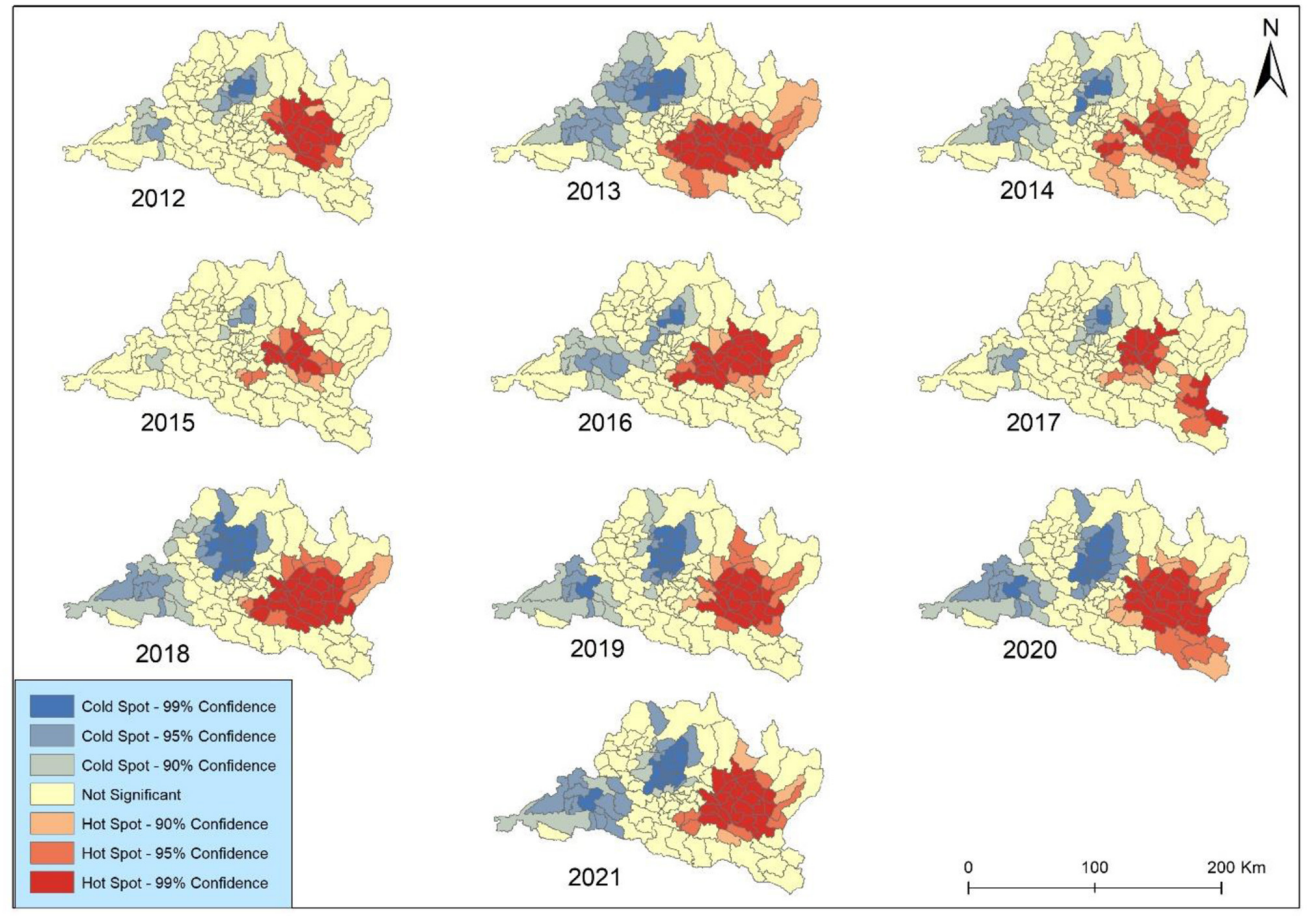
Hotspot analysis of lung cancer incidence in Bagmati province.

The results of Getis-Ord Gi^*^ spatial statistic were presented to identify concentration trends in lung cancer incidence in Bagmati province (Figure 7). The focal points were primarily clustered within the complex landscape in the vicinity of the Kabhrepalanchok, Bhaktapur, Sindhupalchok, and Ramechhap districts. The analysis revealed spatiotemporal variations in hotspot development over the study period. The coldspot was mainly observed around Chitawan, Makawanpur, and Rasuwa throughout the study periods. Notably, no significant geographical concentrations of either higher hotspots or coldspots were observed across Kathmandu and Lalitpur metropolitan cities after 2019. However, consistent coldspots were identified in Bharatpur metropolitan city each year. All identified coldspots exhibited 99 and 95% confidence levels.

The hot and cold spots experienced a minor contraction in 2 years (2012 and 2019) but expanded in the remaining 8 years. The coldspot was surrounded by secondary-level all year except in 2012. The hotspot exhibited elevated occurrences in 2013, 2016, and 2018 across all municipalities in Kabhrepalanchok and most municipalities in Sindhupalchwok and Ramechhap. Notably, Barhabishe municipality demonstrated a pronounced concentration pattern and a rise in lung cancer cases despite being categorized as a region with a lower health risk (very low) throughout the study period.

**Figure 8:**
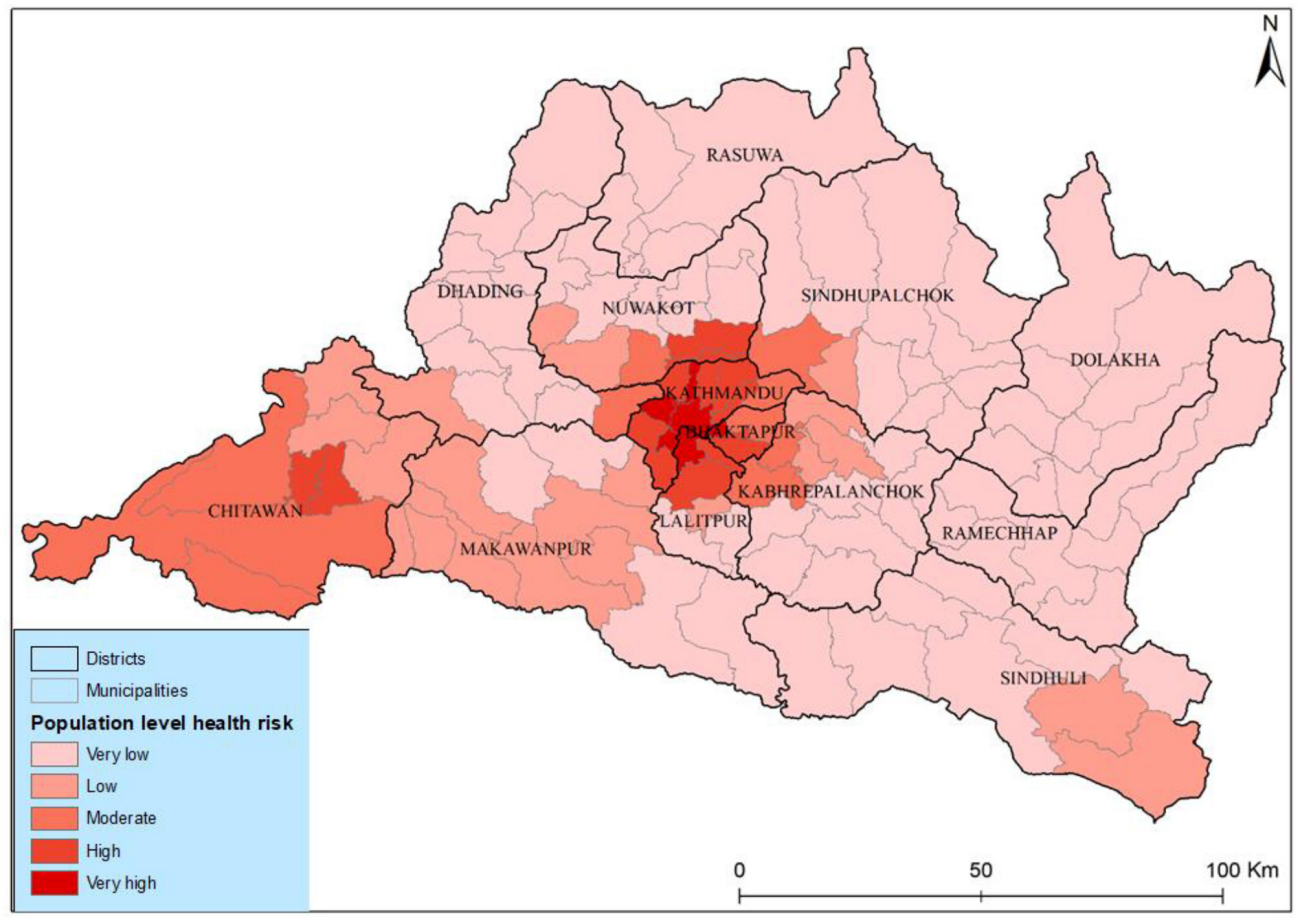
Spatial distribution of lung cancer risk based on a spatially weighted sum.

The results of the population-level health risk assessment show a very high risk in the three municipalities' different districts of Kathmandu Valley. The risk level coincided with the geographical concentration of both populations and PM_2.5_ in these areas as illustrated in Figure 8. Medium-level health risks were identified in municipalities such as Tokha, Kageshwori Manohara, Kirtipur, Mahalaxmi, and Bharatpur. Conversely, municipalities in Rasuwa, Dolakha, and Ramexap districts exhibited the lowest health risks. The Shailung, Doramba, And Ruby Valley of Bagmati province experienced the lowest health risks (very low) from PM_2.5_. The observed spatiotemporal variations in health risk within Bagmati province, particularly in Kathmandu Valley, indicated a concerning increasing trend.

The correlation matrix (Table 2) illustrates the spatial correlation analysis conducted from 2012 to 2021, which showed strong positive correlations among the annual incidence rates, indicating consistent spatial patterns in lung cancer prevalence over the study period. Specifically, correlations between successive years demonstrate high levels of consistency, with coefficients ranging from 0.712 to 0.925. This suggests a persistent spatial autocorrelation in lung cancer incidence rates, indicating that areas with high rates tend to remain clustered together over time. For instance, correlations were notably high in 2014 (0.913) and 2016 (0.925). The correlation between PM_2.5_ and lung cancer is relatively weak, with coefficients ranging from 0.125 in 2016 to 0.195 in 2018. This suggests a limited direct association between fine particulate matter pollution and lung cancer incidence. However, the spatial correlation between lung cancer incidence and PM_2.5_ concentrations in different municipalities was spatially distributed. Overall, the correlation pattern highlights the importance of considering both temporal trends and spatial relationships in understanding the dynamics of lung cancer incidence and its potential environmental determinants.

**Table 2:** Correlation matrix of the spatial correlation analysis among annual lung cancer incidence rates between years 2012–2021.

	**2012**	**2013**	**2014**	**2015**	**2016**	**2017**	**2018**	**2019**	**2020**	**2021**	**PM_2.5_**
2012	1										
2013	0.903	1									
2014	0.91	0.892	1								
2015	0.821	0.796	0.913	1							
2016	0.828	0.823	0.91	0.925	1						
2017	0.777	0.796	0.811	0.848	0.865	1					
2018	0.881	0.888	0.914	0.856	0.871	0.803	1				
2019	0.767	0.678	0.78	0.724	0.756	0.617	0.757	1			
2020	0.774	0.686	0.76	0.701	0.749	0.619	0.758	0.876	1		
2021	0.729	0.725	0.73	0.683	0.745	0.673	0.717	0.779	0.712	1	
PM_2.5_	0.163	0.152	0.162	0.125	0.136	0.183	0.195	0.158	0.177	0.162	1

The geographically weighted regression GWR model is the advancement of the classical linear regression model, which means the ArcGIS adds several spatial analysis methods on the system, it adds the geographic coordinates into the classical linear regression model (29). In the study, before performing the GWR, we applied ordinary linear regression, to confirm the rationality of selecting the index from the extended GWR model. We applied the ordinary least square (OLS) method to validate the results. The *R*^2^ and adjusted *R*^2^ indicate the model fitting is better. The estimated coefficients of PM_2.5_ indicated lung cancer incidence and PM_2.5_ are significantly and positively related (*B* = 0.0387, *P* < 0.05). Therefore, from the OLS, it could be validated that the PM_2.5_ variables are reasonable, and we could continue to perform GWR. The GWR model best fits where values range from 0.5 to −0.5 values. Beyond this, the local GWR models were poorly fitted. Overall, the GWR analysis provides valuable insights into the localized relationships between explanatory variables and lung cancer incidence rates, contributing to our understanding of spatially varying risk factors and informing targeted intervention strategies.

**Figure 9:**
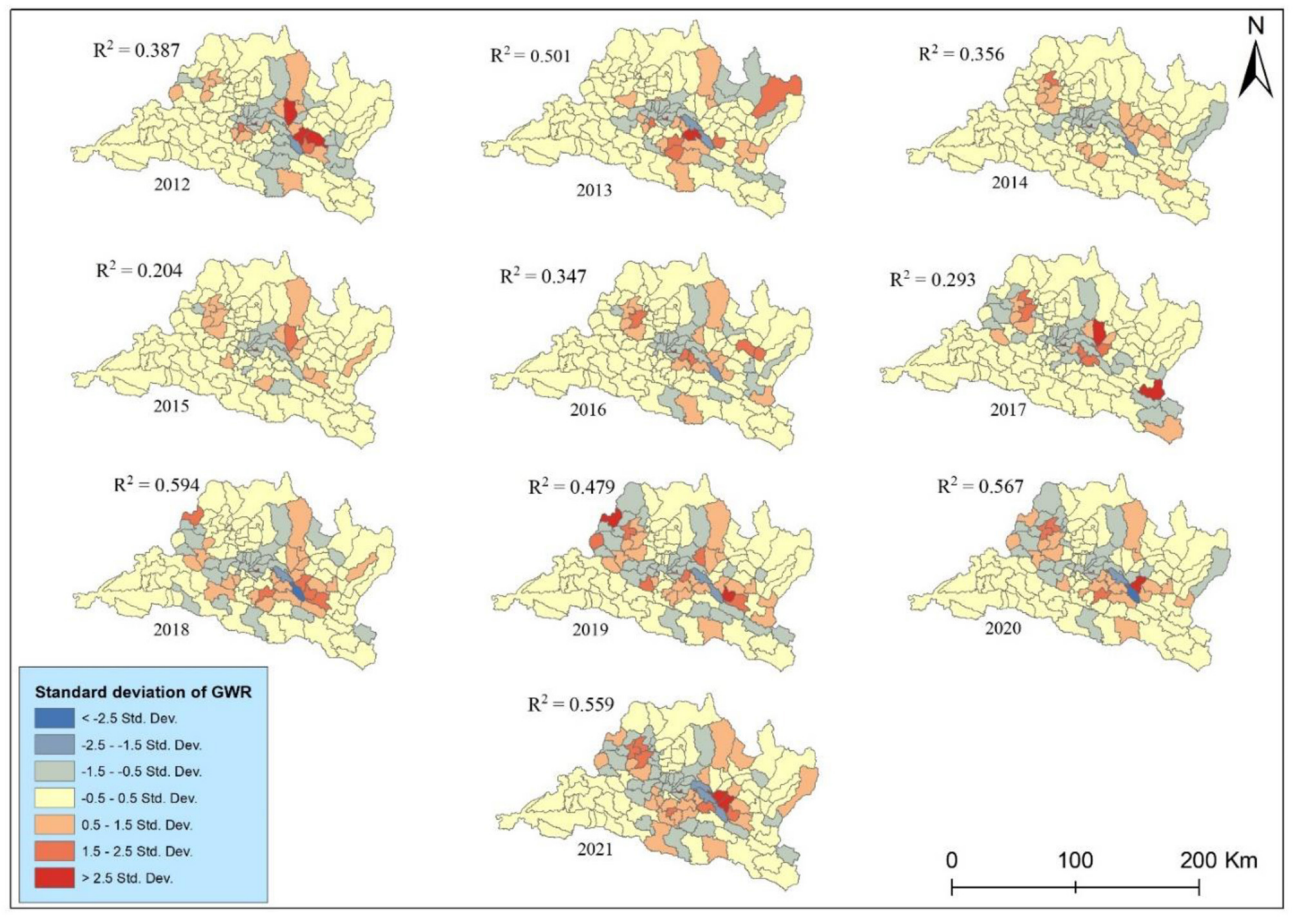
Geographically-weighted regression.

The GWR analysis between cancer incidence and PM_2.5_ showed a fluctuating pattern of *R*-squared over the years (Figure 9). The R-squared values indicate the strength of the relationship between explanatory variables (PM_2.5_) and lung cancer incidence rates. The highest r squared was observed in 0.594 in 2018 indicating a period when PM_2.5_ exposure had a strong association with lung cancer risk. The result explains nearly 60% of the variance, while the GWR shows the lowest association between them in 2015. Conversely, the lowest *R*^2^ value of 0.204 in 2015 suggests a substantial decrease in the predictive power of PM_2.5_. The GWR analysis of lung cancer risk associated with PM_2.5_ in the study area during the study periods presents significant temporal variability in the strength of this association. The *R*^2^ values, representing the proportion of variance in lung cancer risk explained by PM_2.5_ levels, exhibit a moderate to strong relationship overall, with notable peaks and troughs. Despite these fluctuations, the generally high *R*^2^ values in recent years, particularly from 2018 to 2021, underscore the persistent and significant influence of PM_2.5_ on lung cancer risk in the region.

## Discussion

This spatial epidemiological study provided updated lung cancer incidence rates (both CIR and SIR) in Bagmati province during the last 10 years based on Bhaktapur Cancer Hospital data. The study calculated and visualized the spatial and temporal distributions of lung cancer incidence and population-level health risks from PM_2.5_ across the Bagmati province. In addition, the study used the space-time geospatial approach to classify municipal-level health risks among the people by overlaying the population density and PM_2.5_ concentrations. We visualized the result in different maps through GeoDa and ArcGIS to show the association, spatial distributions, and temporal variation of lung cancer in the study area.

Our comprehensive analysis spanning a decade presents a detailed examination of the municipal-level spatial distribution patterns of lung cancer in Bagmati province. As illustrated in Table 1, lung cancer incidence rates reveal notable variations across different years. The standardized incidence rate peaked in 2019, indicating a heightened disease burden. Increased burden of cancer has also been reported in other recent studies. For example, National Health Research Council (NHRC), reported an incidence rate of 41.6 per 100,000 population (10). The age-standardized incidence rate for lung cancer in Nepal is reported at 12.3 per 100,000 population, surpassing incidence rates observed in neighboring countries like India yet lagging behind the higher rates documented in China (8). GLOBOCAN report (2022) highlights the risk of lung cancer, noting 2,431 new cases with an SIR of 9.3. Furthermore, research by Jha et al. (30) delineates a wide-ranging incidence rate for lung cancer in Nepal, spanning from 2.6 to 23.7 per 100,000 across diverse geographic regions of Nepal.

This study presents a detailed examination of lung cancer incidence in both genders. In males, there was an overall increasing trend in cancer incidence, but a notable reversal occurred after 2012, indicating a potential shift in the epidemiological landscape. In contrast, females exhibited a fluctuating trend from 2014 to 2016, showcasing periodic variations in cancer incidence. This observation contrasts with previous studies by Pun et al. (11) and Shrestha et al. (31), which also reported escalating cancer trends in Nepal. Recent studies in Nepal also reported the increasing trend of cancer in both males and females from 2003 to 2012. The study by Poudel et al. (9) showed that lung cancer was the major cancer throughout the last decade in males. A study in Nepal reported the same result; according to the study, lung cancer is increasing in urban areas of Nepal (11, 32). It was because of high pollution concentration in urban areas (33), household air pollution (34), unhealthy food, sophisticated lifestyle, tobacco consumption (8, 35), and insufficient medical health education (36). Furthermore, during the study period, the crude and standardized incidence rates of lung cancer in the study area increased from 2012 to 2016 but decreased in 2017 and 2020. Among all the municipalities of Bagmati province, Panchkhal, Bhumlu, Chautara-Samachokgadhi, Sunapati, and Ramechhap have an increased risk of lung cancer.

Nepal had the highest age-specific lung cancer incidence rate in males during 2013–2015, among other cancers (31). These results also show that the risk factor of lung cancer is still not decreasing and has huge problematic risk characteristics that have not changed. The incidence rate in Bhaktapur and Panchkhal municipalities increased nearly three times from 2012 to 2021. The high risk in these municipalities might be due to screening and a high air pollution concentration. Several possible reasons may account for this phenomenon, including (1) accumulation of lung cancer from PM_2.5_ because of the geospatial attributes (37) and (2) increased urban areas and pesticide trade within a study area as part of the development activities and increase of agricultural production in Bagmati province (38, 39). A noteworthy observation is the consistent elevation of the incidence rate in males in urban areas. During this study period, the incidence rate in females in rural municipalities surpassed that of males. The lung cancer incidence exhibited analogous patterns in both genders within Bagmati province. However, a substantial incongruity emerged in the urban areas, where the LC was approximately twice that of rural areas, highlighting significant rural-urban variations.

The spatial autocorrelation analysis was performed PM_2.5_ and Spatial Empirical Bayes smoothed incidence rate using Moran's I index. This metric ranges from −1 to +1, where −1 indicates perfect negative spatial autocorrelation, +1 indicates perfect positive spatial autocorrelation, and 0 denotes a spatially random distribution (40). Our study revealed that Moran's I values for lung cancer risk were consistently higher than 0 in the study area, indicating the presence of spatial clustering in Bagmati province (Figure 7). This suggests a significant clustering of lung cancer risk in this province, highlighting the importance of further investigation into potential environmental or demographic factors contributing to this clustering phenomenon.

The lung cancer distribution has been attributed to population density, diverse topography, and pollution to lung cancer (23, 41, 42), most likely linked to pollution exposure (PM_2.5_) and the prevalence of risk in Bagmati province. PM_2.5_ is heterogeneously distributed; it has a high concentration in the Kathmandu Valley and Chure Siwalik region of Bagmati province. The study presented the same population distribution pattern (Figure 1). Kathmandu Valley has the highest population density. Thus, analyzing the municipal-level distribution of lung cancer risk provides a comprehensive understanding and helps to focus on the prevention strategy.

The hotspot experienced a continuous expansion from 2012 to 2018 but slightly shrinkage in 2019. Zhang and Tripathi (26) found that two regions (Chonburi and Chanthaburi) of Eastern Thailand were the major hot spots for lung cancer risk, situated near industrial areas. Our study identified the heterogeneous hotspots within Bagmati province; however, some municipalities like Bhaktapur and Panchkhal were identified as major HH clusters during the study periods. These areas are attributed to high concentrations of PM_2.5_ and pollution accumulation (37). This study highlights that it is important for effectively controlling lung cancer risk. Policymakers and healthcare authorities can strategically allocate resources and implement targeted intervention strategies by pinpointing these areas with heightened risk. This approach enables focused efforts to mitigate the impact of lung cancer in high-risk regions, thereby optimizing resource utilization and potentially improving health outcomes for affected populations (22, 43). Overall, identifying these hotspots is a valuable tool in guiding proactive measures aimed at reducing the burden of lung cancer within Bagmati province and enhancing the effectiveness of public health initiatives in combating this disease.

Incorporating municipal population density and PM_2.5_ into our analysis generated a comprehensive risk assessment. The population-level health risks attributed to PM_2.5_ are high for those who live in core urban areas with high concentrations of pollution, showing very high health risks in core city areas and high health risks in peripheral areas of Kathmandu Valley and Chitawan. The Kabhrepalanchwok, Nuwakot, and Ramechhap municipalities witnessed a significantly moderate concentration pattern and increased lung cancer risk. However, it was defined as a lower health risk area. This approach enhances the accuracy of identifying risk zones and provides valuable insights for targeted mitigation efforts. Our weighted sum overlay analysis offers a practical tool for prioritizing resource allocation and intervention strategies in high-risk areas by leveraging the strong evidence linking population density to lung cancer prevalence. Ultimately, it empowers policymakers and healthcare professionals with the information needed to address the burden of lung cancer within Bagmati province proactively.

Previous research has indicated that lung cancer and cardiovascular disease are associated with environmental factors, geographical location, and topography (23, 44). The study also presented spatiotemporal variations of lung cancer risk in Bagmati province associated with environmental factors (PM_2.5_) at the municipal level. The overall temporal distribution of lung cancer incidence rate is significant, but spatial correlations between lung cancer and PM_2.5_ distributions were not strongly significant. However, the study found differences at the municipal level compared to the Bagmati province. Furthermore, our study highlights the spatial heterogeneity in lung cancer risk at the municipal level across the study periods. Geographically weighted regression models can potentially enhance the explanatory power of risk factors to demonstrate spatial variations in lung cancer risk (23). This model can present the association between environmental variables and lung cancer risk, which will be effective in running the lung cancer intervention program in Bagmati province.

The study found a fluctuating temporal correlation in the study area between 2012 and 2021. The study investigated the spatial relationship between the distribution of PM_2.5_ and the incidence rate of lung cancer across the municipal level of Bagmati province using the GWR model. The findings indicate a noteworthy spatial association between PM_2.5_ and lung cancer, particularly in geographically concentrated areas with very high-risk municipalities. The risks of lung cancer incidence associated with fine particulate matter. Results show that PM_2.5_ emissions and concentrations have similar distribution patterns: high values existed in the urban areas with high population density and incidence. In contrast, the village area had a low incidence rate. These results underscore the potential impact of pollution activities on public health outcomes, emphasizing the need for targeted interventions and further investigation into mitigating factors in affected municipalities.

This research significantly enhances our understanding and contribution to the field of lung cancer and spatial epidemiology in Nepal by adding the spatiotemporal approach to the correlation with environmental and geographical perspectives. Adopting a health geography perspective to explore a smaller area's fundamental modifiable areal unit problem (MAUP) was much more effective in analyzing lung cancer (22, 45). This study strategically employed MAUP to study the municipality as the unit of analysis, providing valuable insights into the intricacies of lung cancer in Bagmati province. It was also an advantage in conducting environmental health-related research. Although socioeconomic status and healthcare accessibility may influence lung cancer, it is noteworthy that these elements were relatively evenly distributed across the study area.

However, this study was completed with several limitations. We acknowledge that a significant limitation of our study is the reliance on lung cancer incidence data from a single institution, Bhaktapur Cancer Hospital (BCH), which represents only a portion of the overall patient population in Bagmati Province and contributes ~20% to the Population-Based Cancer Registry (PBCR) system in Nepal (62). Bagmati Province has multiple institutions providing cancer care, and while including data from all hospitals would ideally yield a more comprehensive sample, access to these datasets was beyond our scope due to resource and logistical constraints.

This single-center approach may have several potential biases. Albeit, it is the leading cancer hospital in Nepal. Selection bias may be minimal due to the patient population at BCH has unique characteristics, such as distinct demographic or socioeconomic factors, that differ from those receiving treatment elsewhere. Referral bias could also impact generalizability, as BCH may treat more advanced or specific cases of lung cancer, characteristic of tertiary-care centers, which can influence the representation of prognostic factors. Additionally, underreporting or over reporting is possible since cases treated at other facilities are not captured, potentially skewing the observed incidence patterns. Lastly, temporal bias may affect data consistency over the study period due to evolving diagnostic criteria, treatment practices, and reporting standards.

Moreover, this study is subject to the modifiable areal unit problem (MAUP), where spatial analysis results can vary based on how geographic units, like municipalities, are defined and their sizes (61). The way municipalities are divided may influence the spatial patterns observed, potentially affecting the strength of associations between PM2.5 exposure and lung cancer risk. Moreover, we do not incorporate topographical and meteorological components to understand the dynamics of lung cancer, representing another limitation in this study. Although topographical phenomena are key influential factors in epidemiology, they contribute highly to lung cancer (46).

Other factors contributed to significantly higher lung cancer incidence in men than women in Bagmati Province, Nepal. Lung cancer risk among men is elevated due to (1). higher smoking rates (3), (2). occupational exposures, particularly in agriculture, construction, and brick manufacturing, significantly increase the risk of lung cancer due to exposure to hazardous chemicals, respirable crystalline silica, and poor working conditions, highlighting the urgent need for improved health and safety standards. Prajapati et al. (47) increased alcohol use, which amplifies tobacco's carcinogenic effects (48). While women face higher indoor air pollution exposure from cooking with solid fuels, traditional household roles and limited healthcare access may delay diagnosis, affecting lung cancer incidence reporting (49). It is important to note that the study results may be biased, potentially underrepresenting or over representing the actual relationships between PM2.5 exposure and lung cancer risk without controlling for these confounding factors. Although this study focuses solely on the association with PM2.5, these additional factors are important considerations in understanding gender disparities in lung cancer risk for future work.

Despite these limitations, our analysis still provides valuable insights into lung cancer trends and spatial associations with PM_2.5_ exposures within Bagmati Province. Using geographically weighted regression (GWR) and spatial autocorrelation analysis, we identified notable hotspots and trends that align with known population and environmental patterns. These findings, while limited to a subset of patients, offer a preliminary understanding that can guide future studies with expanded regional data access, potentially enhancing the generalizability of conclusions.

## Conclusions

This study analyzed municipal-level lung cancer incidence in Bagmati province and explored the potential association with PM_2.5_. The result demonstrated a significant difference in the risk of lung cancer over the age and gender where male and older adult populations were more likely to get lung cancer compared with female and young age people. In terms of spatiotemporal distribution, marked spatial variations and heterogeneity were observed. The statistically significant hotspots were detected over the years. The heterogeneous association between lung cancer incidence and PM_2.5_ was observed and remained changing over the years. The results further showed the risk of lung cancer is increasing over the years. The findings revealed a concentrated pattern of environmental risks and health outcomes around the capital city, with notable spatiotemporal variations.

The government needs substantial information about the spatiotemporal distribution of LC to run an effective strategy for LC prevention. The spatiotemporal studies must be associated with several characteristics of environmental factors like PM_2.5_. In general, the results of this study are of great significance to public health issues in Nepal. The temporal and spatial analysis of lung cancer incidence provides important epidemiological clues for the investigation mechanism of lung cancer. It provides a scientific basis for the rational allocation of health resources. Based on these findings, it is important for mass early screening among people in high-risk areas of the study areas. Spatial information on diseases, as well as lung cancer, in Nepal is limited. This study can be the pathway of new research in Nepal under the geo-health theme.

## Data Availability

The datasets presented in this study can be found in online repositories. The names of the repository/repositories and accession number(s) can be found in the article/supplementary material.
